# A seed-expanding method based on random walks for community detection in networks with ambiguous community structures

**DOI:** 10.1038/srep41830

**Published:** 2017-02-03

**Authors:** Yansen Su, Bangju Wang, Xingyi Zhang

**Affiliations:** 1 Key Lab of Intelligent Computing and Signal Processing of Ministry of Education, School of Computer Science and Technology, Anhui University, Hefei 230039, China

## Abstract

Community detection has received a great deal of attention, since it could help to reveal the useful information hidden in complex networks. Although most previous modularity-based and local modularity-based community detection algorithms could detect strong communities, they may fail to exactly detect several weak communities. In this work, we define a network with clear or ambiguous community structures based on the types of its communities. A seed-expanding method based on random walks is proposed to detect communities for networks, especially for the networks with ambiguous community structures. We identify local maximum degree nodes, and detect seed communities in a network. Then, the probability of a node belonging to each community is calculated based on the total probability model and random walks, and each community is expanded by repeatedly adding the node which is most likely to belong to it. Finally, we use the community optimization method to ensure that each node is in a community. Experimental results on both computer-generated and real-world networks demonstrate that the quality of the communities detected by the proposed algorithm is superior to the- state-of-the-art algorithms in the networks with ambiguous community structures.

Extensive researches on real-world networks show that community structure is an important property^1^,^2^. The nodes of the same community may be the individuals with certain relationships in social networks, the genes or proteins with the similar function and the web pages dealing with the same topic^3^,^4^. Therefore, it is helpful to reveal community structures to understand the structures of networks and detect potentially useful information of networks.

Communities can be loosely defined as the subsets of nodes which are more densely linked than the rest of the network. In this sense, modularity and local modularity were proposed as indices of community structure^1^,^5^. There are also two community definitions (i.e., the strong and weak community definitions) based on the topology of networks, where the strong community is the community in which each node has more connections than in each rest community and the weak community is the community in which the sum of all degrees is larger than the sum of all degrees in each rest community^6^,^7^. In this article, following the strong and weak community definitions, we define that a network has a clear or ambiguous community structure as follows. If the communities in a network are all strong communities, then the network has a clear community structure; otherwise, if some communities in a network are weak communities, then the network has an ambiguous community structure.

Many efforts have focused on detecting community structures in complex networks^8^,^9^,^10^,^11^,^12^,^13^,^14^. Popular algorithms include modularity-based algorithms (e.g. Girvan-Newman algorithm (*GN*)^8^, fast Newman algorithm (*FN*)^9^ and Fast unfolding algorithm (*FUA*)^13^) and local modularity-based algorithms (e.g. local maximum degree algorithm (*LMDR*)^14^). Most of the modularity-based and local modularity-based community detection algorithms could exactly detect communities in several networks, especially for the networks with clear community structures^15^,^16^,^17^. However, these algorithms still suffer from some limitations, which may prevent them to achieve satisfactory performance on the network with ambiguous community structures. For instance, the modularity-based measurement may fail to identify the modules which are smaller than the scale which depends on the total number of edges and on the degree of interconnectedness of the modules^7^. *LMDR* is a greedy maximum algorithm which starts from a local degree central node whose degree is greater than or equal to the degree of its neighbor nodes, and then iteratively adds the nodes yielding the largest increase of the local modularity until the community reached a predefined size. However, some weak communities may fail to be detected by *LMDR*. The modularity-based and local modularity-based algorithms mainly maximize the modularity and local modularity, which only compare the inner edges of a community with the edges between the community and the rest part of the network. Thus, it is hard to exactly detect some weak communities by the modularity-based and local modularity-based algorithms.

Besides the community detection methods mentioned above, researches explore several random walks-based methods for community detection (e.g., a seed set expansion algorithm^18^ and an algorithm for finding and extracting a community (*FEC*)^19^), since the random walks-based techniques have a good ability to deal with uncertainty or fuzziness. Previous researches show that the communities identified by random walks-based algorithms are structurally close to real-world communities^20^. Specifically, the basic idea of *FEC* algorithm is that a random walker is more likely to reach the nodes in its own community, when compared to other communities^19^. Following the basic idea, the algorithm checks whether a node should be added into the community by comparing the probability of this node in the community with the one of the node in each of the rest communities. It is likely to identify the communities, in which each node has more connections than in each rest community. However, *FEC* is unstable, as the algorithm starts with an arbitrary destination node; the performance of *FEC* needs to be enhanced in networks, as it is hard to accurately detect weak communities.

Here, inspired by the basic idea of *FEC* algorithm, we propose a random walks-based algorithm named *RWA* to detect communities for complex networks, especially for the networks with ambiguous community structures. The overall framework of *RWA* is selecting the dense subgraphs which contain important nodes in a network, and expanding these dense subgraphs based on random walks. Specifically, (1) the seed communities are detected based on the nodes whose degree is greater than or equal to the degree of its neighbor nodes; (2) the seed communities are expanded using random walks; (3) the expanded communities are adjusted to ensure each node in a network is in a community. A difference between *FEC* and *RWA* is that, a seed in *FEC* is an arbitrary node which leads to the instability of detection results, while a seed in *RWA* is a dense subgraph which could avoid the instability of detection results. The performance of *RWA* is tested on both computer-generated and real-world networks. Experimental results demonstrate that the quality of the communities detected by *RWA* is superior to those detected by comparative algorithms, especially in the networks which have ambiguous community structures. *RWA* may be helpful to understand the real-world networks, most of which have ambiguous community structures.

## Results

This section presents the comparative results of the proposed algorithm and the traditional algorithms in the experiments preformed on both computer-generated and real-world networks.

### Computer-generated and real-world networks

The first kind of computer-generated networks employed in the experiments are the GN benchmark networks, proposed by Girvan and Newman^8^. This network is constructed as follows: 128 nodes are randomly and equally divided into four communities; edges are randomly placed between node pairs to make the average degree of the graph equal to 16. Each pair of nodes in the same community has an edge with probability *P*_*in*_. Here, *P*_*in*_ is a parameter of networks generated. Generally speaking, when *P*_*in*_ < 0.40, it is unable to detect community structures. When the value of *P*_*in*_ becomes larger, the community can be more easily detected. In our experiments, 0.40 ≤ *P*_*in*_ ≤ 0.90. For each 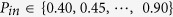, 100 networks are generated. According to the parameter *P*_*in*_, the computer-generated networks could be classified into two classes. When 0.80 ≤ *P*_*in*_ ≤ 0.90, all of the communities in the networks are strong communities (p-value = 0.05). These networks have clear community structures. When 0.40 ≤ *P*_*in*_ < 0.80, some of the predefined communities are not strong communities, but they are weak communities. In this situation, the networks have ambiguous community structures.

Another set of computer-generated networks is the LFR benchmark networks^21^. Compared with the GN benchmark networks, the LFR benchmark networks have more adjustable parameters, which control the number of nodes generated, the average degree of nodes and the size of communities generated. The LFR benchmark networks mainly include the following parameters: *N* is the number of nodes in networks; *d* is the average degree of nodes in network; *Maxd* is the biggest degree of node; *Minc* is the number of nodes that the smallest community contains; *Maxc* is the number of nodes that the biggest community contains; and *μ* is the probability of nodes connected with nodes of external community. The bigger *μ* is, the more difficult the community detection is. When *u* ≥ 0.3, the networks have ambiguous community structures (p-value < 0.05). We produce two groups of the LFR benchmark networks. These two groups share these parameters *d* = 10, *Maxd* = 50, *Minc* = 10 and *Maxc* = 20. The numbers of nodes in these two groups of networks are set to *N* = 200 and *N* = 300, respectively. The value of *μ* in each group is set from 0.1 to 0.6, with the interval 0.1.

We also employ four real-world networks in the experiments. The four real-world networks are the Zachary’s Karate Club network (Karate network, for short)^22^, the Bottlenose Dolphins network (Dolphins network, for short)^23^, the Books about US politics network (Polbooks network, for short)^24^ and the American College Football network (Football network, for short)^8^, respectively. Each real-world network employed in our work has at least one weak community (see Table 1). Thus, all of the four real-world networks have ambiguous community structures.

### Comparison and evaluation

#### Comparison with other algorithms

We verify the performance of the proposed algorithm (*RWA*) by comparing it with five representative algorithms (*GN, FN, FUA, FEC* and *LMDR*) on both computer-generated networks and four real-world networks.

**Figure 1:**
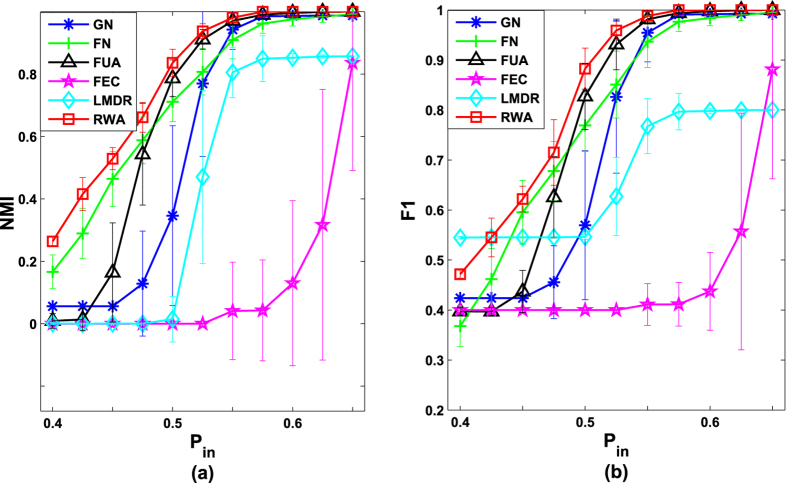
The comparative results on the GN benchmark networks. Each point is the mean of *NMI* and *F*1 values averaged over 30 independent runs. Error bars show the standard deviations estimated from 30 networks.

**Figure 2:**
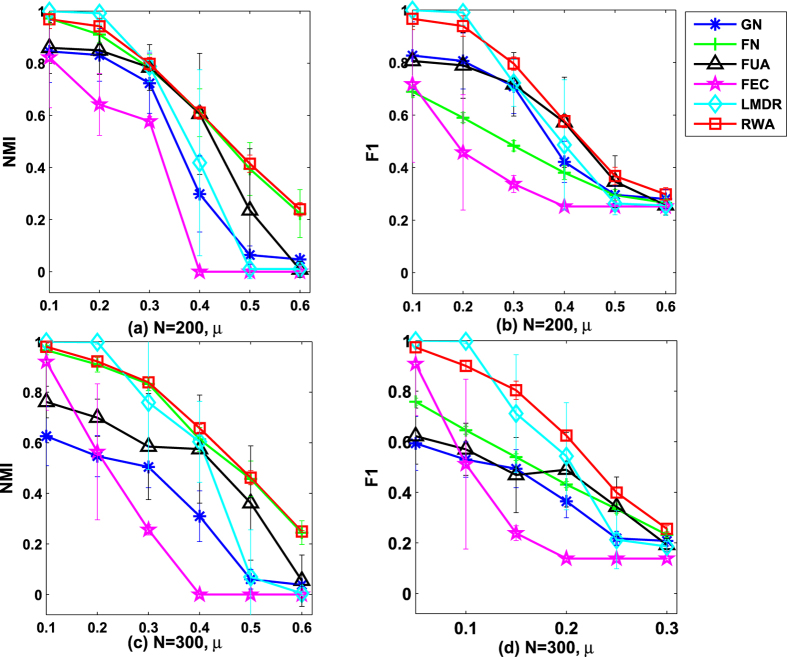
The comparative results on the LFR benchmark networks. Each point is the mean of *NMI* and *F*1 values averaged over 30 independent runs. Error bars show the standard deviations estimated from 30 networks.

**Figure 3:**
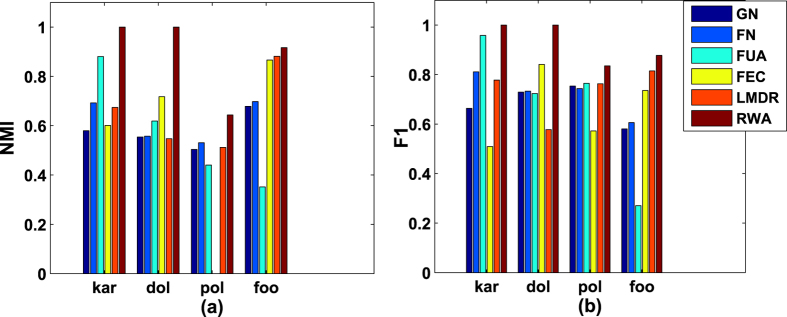
The comparative results on real-world networks. ‘kar’, ‘dol’, ‘pol’ and ‘foo’ represent the Zachary’s Karate Club network, the Bottlenose Dolphins network, the Books about US politics network and the American College Football network, respectively.

1. We apply our algorithm and other five algorithms (*GN, FN, FUA, FEC* and *LMDR*) to the GN benchmark networks with 128 nodes and four predetermined communities 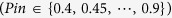. The comparative results on computer-generated networks are given in Fig. 1, with both the mean of the normalized mutual information (*NMI*) values and the mean of the F-measure (*F*1) values averaged over 30 independent runs for *RWA* and other five representative algorithms.As can been seen from Fig. 1(a), when the networks have clear community structures (i.e., *P*_*in*_ ≥ 0.80), all algorithms except *FEC* and *LMDR* can get the nearly true partition results (*NMI* value is nearly 1.0). In this situation, *RWA* performs very similar to the comparative algorithms (*GN, FN* and *FUA*). However, *RWA* generates the best detection results when the networks have ambiguous community structures (0.40 ≤ *P*_*in*_ < 0.80), and the results obtained through our algorithm remain relatively stable. When the networks have clear community structures (i.e., *P*_*in*_ ≥ 0.80), the *F*1 value obtained through *RWA* is no less than those obtained by other five comparative algorithms. In details, the *F*1 values of *RWA* and four comparative algorithms (*GN, FN* and *FUA*) are almost 1.0 when *P*_*in*_ ≥ 0.80. That is, the proposed algorithm and four of the five comparative algorithms could get the nearly true partition results. In contrast, both *LMDR* and *FEC* produce the *F*1 values which are less than 0.90. When the networks have ambiguous community structures (i.e., 0.40 ≤ *P*_*in*_ < 0.80), the values of *F*1 obtained by *RWA* are not the largest, and *RWA* performs slightly less well than some of the comparative algorithms (e.g. *LMDR*) for few detection problems (i.e., *P*_*in*_ = 0.40). However, the detection results shows that *RWA* has the best performance. When two evaluation measures (*NMI* and *F*1) are considered together, although the *F*1 value of *RWA* is smaller than that of *LMDR* for few detection problems, the performance of *RWA* is still better than *LMDR*. Actually, when 0.40 ≤ *P*_*in*_ ≤ 0.55, it can be seen that the *F*1 values of *LMDR* are lager than some comparative algorithms, and the *NMI* values of *LMDR* are equal to zero in networks. That is because in these situations, all nodes in the network fall into a community, which is far from the true partition. Besides, the *F*1 values obtained through *RWA* decline relatively stable, and our algorithm obtains the best results when 0.4 ≤ *P*_*in*_ < 0.80. We can conclude from Fig. 1 that *RWA* performs the best among the comparative algorithms on the GN benchmark networks, especially when the networks have ambiguous community structures.
2. In our work, the performance of *RWA* is also compared with other five algorithms on two groups of the LFR benchmark networks. Figure 2 shows the average results over 30 runs on LFR benchmark networks. It can be seen form Fig. 2(a,c) that, when the value of *μ* is smaller than or equal to 0.2, the *NMI* obtained by *RWA* is larger than 0.9, but it is less well than *LMDR*. It suggests that although *RWA* gets the nearly true partition results, its performance is not the best. As *μ* increases, the *NMI* obtained by *RWA* remains relatively stable and *RWA* obtains the best results when *μ* is greater than or equal to 0.3. Similarly, *RWA* generates the largest and stablest value of *F*1 when *μ* is larger than or equal to 0.3 (see Fig. 2(b,d)). The value of *NMI* obtained by *RWA* is a slightly larger than that obtained by *FN*. However, compared with *FN, RWA* generates much larger value of *F*1. It is concluded that the performance of *RWA* is superior to the comparative algorithm on the LFR benchmark networks.
3. All algorithms run 30 times on the four real-world networks, and the average *NMI* values and the average *F*1 values are shown in Fig. 3. As can be seen from Fig. 3(a), *RWA* generates significantly better results than the comparative algorithms. Specifically, *RWA* can achieve the largest *NMI* values on the four real-world networks (*p*_*value* < 0.05). Similarly, as can be seen from Fig. 3(b), the average *F*1 values obtained by *RWA* are also larger than the comparative algorithms on the four real-world networks (*p*_*value* < 0.05). Therefore, the proposed algorithm achieves the best detection results when tested on the four benchmark networks.

The communities in a real-world network could be divided into two classes: strong and weak communities. For each class, we count the number of times that each algorithm shows the best performance. As we can see from Table 2, one of the comparative algorithms shows better performance than the proposed algorithm in ≤60% of all strong communities and ≤22.22% of all weak communities. However, in 60% of all strong communities and 77.78% of all weak communities, *RWA* shows the best performance. That is, *RWA* surpasses previously proposed algorithms in most cases. We can conclude that *RWA* performs better than the comparative algorithms in both strong and weak communities, particularly in weak communities.

#### Selection of the parameter *Z*

In the proposed algorithm, a seed community is used as a seed by extending it to a larger community. The nodes of the seed community should be connected as densely as possible. To this end, we choose a complete subgraph as a seed community. Due to the fact that a complete subgraph with one node or two nodes is meaningless, we only consider complete subgraphs consisting of three or more nodes in this work. Let *Z* be the number of nodes in a seed community. In the following, we investigate the influence of *Z* ≥ 3 on the performance of *RWA* in our work.

**Figure 4:**
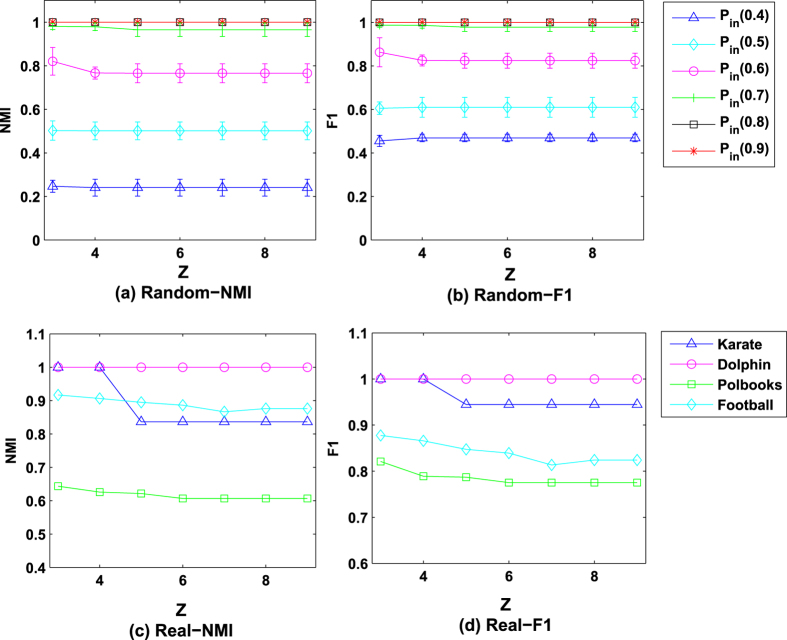
Sensitivity of *Z*. Each point is the mean of *NMI* and *F*1 values averaged over 30 independent runs and error bars show the standard deviations estimated from 30 networks.

We do experiments on computer-generated and real-word networks. The results of *NMI* values and *F*1 values for different settings of *Z* 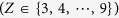 on computer-generated networks 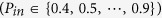 are shown in Fig. 4(a,b), averaging over 30 independent runs. According to Fig. 4(a), if *P*_*in*_ is either 0.6 or 0.7, then the value of *NMI* is the largest when *Z* is 3, and it is little larger than those when *Z* ∈ {4, 5, 6, 7, 8, 9}; if *P*_*in*_ ∈ {0.4, 0.5, 0.8, 0.9}, the values of *NMI* are the same, regardless of what *Z* is. Similarly, if *P*_*in*_ is either 0.6 or 0.7, when *Z* is 3, our algorithm produces the largest *F*1; otherwise, the value of *F*1 is unrelated with *Z*. Thus, the values of *NMI* and *F*1 have low sensitivity of *Z* when the experiments are tested on computer-generated networks. Figure 4(c,d) show the results of *NMI* values and *F*1 values for different settings of *Z* 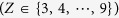 on four real-word networks. We can see that, on Dolphin network, the values of *NMI (F*1) are the same, regardless of what *Z* is; and on Karate, Polbooks, and Football networks, the best performance has been achieved when *Z* is 3.

Sensitivity analysis of this parameter shown in Fig. 4 has indicated that complete subgraphs with three nodes can achieve the best performance of the proposed algorithm. To obtain the best performance of *RWA, Z* can be set to 3.

## Discussion

In this paper, we have proposed the algorithm *RWA* to detect community structure in a network, especially for the network with ambiguous community structure. In order to avoid the instability of detection results, seed communities were detected based on local maximal degree nodes, which have relatively high degree compared with their neighbors. In addition, the seed communities were expanded through random walks by adding nodes step by step.

We have test the performance of the proposed algorithm, and compared it with other representative algorithms on both computer-generated and real-world networks. (1) The experimental results have demonstrated the superior performance of *RWA* over the comparative algorithms (*GN, FN, FUA, FEC* and *LMDR*) in terms of *NMI* and *F*1 for detecting communities. An interesting observation was that the proposed algorithm surpassed five previously proposed algorithms in detecting weak communities in real-world networks. It is concluded that the performance of *RWA* showed more advantages in the networks which have ambiguous community structures, when compared with the comparative algorithms. (2) An initial community is a dense subgraph with *Z* nodes. The experimental results have demonstrated that the proposed algorithm showed good performance with low sensitivity of *Z*. Furthermore, if *Z* is equal to three, then the proposed algorithm gained the best results. Therefore, we adopt *Z* = 3 in our work. In total, the experimental results have showed the effectiveness and robustness of the proposed algorithm. These experimental results confirmed that the proposed algorithm might be more suitable for the community detection of the complex networks with ambiguous community structures.

In future research, we will focus on the detection problem in networks with larger scale, such as networks with hundreds of thousands, or even millions nodes. We will extend the algorithm to detect overlap communities. In addition, we will improve the detection accuracy, so that the algorithm can detect community structures efficiently.

## Methods

The proposed algorithm (*RWA*) aims to select the dense subgraphs which contain important nodes in the network, and expand these dense subgraphs based on random walks. The overall framework of the proposed algorithm (*RWA*) contains the following three steps: (1) A procedure is proposed to detect seed communities based on local maximal degree nodes. These local maximal degree nodes have relatively high degree compared with their neighbors and locate dispersedly in the network, which could be considered as a local hub of a community^14^. (2) A strategy is applied to expand seed communities using random walks. In the expansion process, we calculate the probability of a node in a community based on random walks, and then add the node to the community which it most likely belongs to. A community may have more than one seed community, so that the expanded communities which have a large number of common nodes are deserved to be merged. (3) The expanded communities are adjusted to ensure each node in a network is in a community. In what follows, we introduce the details about *RWA*.

### Detecting seed communities

The basic idea of seed-based community detection algorithms includes the identification of the seeds, which are special nodes in networks^25^. From a topological point of view, a single seed may be a set of nodes which are not necessarily connected^18^,^26^, or a set of nodes which are closely connected^27^. For instance, the seed is proposed to be random nodes in a network^28^. However, it does not use the topological information of the real-world networks. Generally speaking, the nodes which suit to constitute a seed are always the important nodes in a network. The seed has been proposed to be composed of the top *k* highest degree nodes, which playing the role of leaders in the network (i.e., the nodes whose removal from the network implies community collapse)^18^,^26^. Besides, the local hubs, such as the nodes with local maximal degree in a network, are selected as seeds^29^,^30^. The seed is also proposed to be a core set, in which the nodes are densely connected based on structural similarity^27^.

Here, a seed community includes the important node which is most likely in a community, as well as the nodes and edges which are closely connected with the important node. Thus, a single seed is no longer a set of nodes, and it is a dense subgraph in a network. In what follows, the important nodes in a network are identified first, and then the dense subgraphs are detected.

A local maximal degree node is defined as a node which has a larger number of edges compared with its neighbors in a network^14^. Here, we identify local maximal degree nodes from all nodes in a complex network. The way to discover local maximal degree nodes from a given starting node was referred in a pervious work^14^.

We detect the dense subgraphs based on the local hub set, which is a union of all local maximal degree nodes in a complex network. For the node (*node*_1_) in the local hub set, we detect its local maximal degree nodes. The node (*node*_1_) and one of its local maximal degree node (*node*_2_) may have a common neighbor node (*node*_3_). A dense subgraph with three nodes is comprised by the nodes *node*_1_, *node*_2_ and *node*_3_, together with the edges among them. In this way, a dense subgraph with more than three nodes may also be detected. We analyze the influence of the number of nodes in a seed community on the performance of the proposed algorithm. Here we choose the dense subgraph with three nodes to be a seed community.

### Expanding seed communities

Let 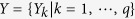 be the set of all communities, where *Y*_*k*_ = (*V*_*k*_, *E*_*k*_) is the *k*^*th*^ community, *V*_*k*_ is the set of nodes in the *k*^*th*^ community, *E*_*k*_ is the set of edges in the *k*^*th*^ community and *q* is the number of communities. Particularly, in the initial situation, 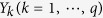 is a seed community.

Let the walker start from a node *u* which does not belong to any communities. The total probability theorem and conditional probability model are used to calculate the probabilities of the walker teleporting from the node *u* to each community (i.e. 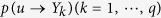). A community is expanded by iteratively adding the nodes which has the largest probability to reach the community. There are *q* communities, so we perform *q* runs of random walks to calculate *p*(*u* → *Y*_*k*_).

At the *k*^*th*^ run of random walks, it is supposed that *u* belongs to the *k*^*th*^ community. The graph of the *k*^*th*^ random walk process is:


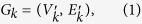


where 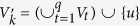, 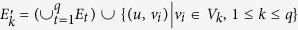.

First, we calculate the probability of the walker teleporting from *u* to the node 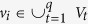 in the graph *G*_*k*_, which is denoted as *p*(*u* → *v*_*i*_|*u* ∈ *G*_*k*_). From the time *t* to the time *t* + 1, the walker has a teleporting probability *α* to jump, as well as a probability 1 − *α* to stay. Usually, the teleporting probability *α* is 0.15^31^. When the walker jumps, it may jump to a node with a transition probability. Suppose that the transition probability for the walker jumping from *u* to each node in 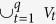 is the same, then the transition probability vector is 
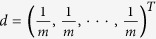
, where *m* is the number of nodes in the node-set 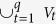 and *d* is a *m* × 1 vector. When the walker stays, it may reach a node on the basis of the similarity between nodes (See the ‘Calculation of similarity’ subsection for the way to calculate the similarity between nodes). Let the matrix *M* with dimension of *m* × *m* denote the normalization of similarity between nodes in *V*. Suppose the probability of the walker teleporting from *u* to *v*_*i*_ is *s*_*t*_(*i*) at the time *t*. At the time *t* + 1, the probability vector *s*_*t*+1_ is calculated as follows.


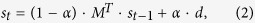


where *M*^*T*^ is the transpose of the matrix *M*, and *t* ≥ 1. Particularly, in the initial situation, the probability of *u* teleporting to *v*_*i*_ is proportional to the similarity between *u* and *v*_*i*_ (See the ‘Calculation of similarity’ subsection). Here, *s*_0_(*i*) is the normalization of the similarity between *u* and *v*_*i*_.

Iterate the Eq. (2) until *s* is convergent. Suppose the distribution vector is *π* = (*π*_1_, …, *π*_*m*_), then *π* satisfies *π* = (1 − *α*) · *M*^*T*^ · *π* + *α* · *d*. In this situation, *π* is the stationary distribution, where the *i*^*th*^ entry captures the conditional probability that the walker teleports from the node *u* to the node *v*_*i*_ when *u* belongs to the *k*^*th*^ community.

Next, the walker has an average conditional probability *p*(*u* → *Y*_*j*_|*u* ∈ *G*_*k*_) to teleport from the node *u* to a community *Y*_*j*_ when *u* belongs to the *k*^*th*^ community. Specifically, *p*(*u* → *Y*_*j*_|*u* ∈ *G*_*k*_) is the average value of the conditional probabilities.


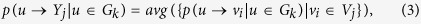


where *p*(*u* → *v*_*i*_|*u* ∈ *G*_*k*_) = *π*_*i*_ and *avg*(*x*) means the average value of the elements in the set *x*.

Finally, the average probability that the node *u* belongs to the *k*^*th*^ community is calculated as:


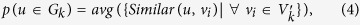


where *Similar*(*u, v*_*i*_) is the similarity between nodes *u* and *v*_*i*_ ∈ *V*_*k*′_ (See the ‘Calculation of similarity’ subsection for the calculation of *Similar*(*u, v*_*i*_)) and *avg*(*x*) means the average value of the elements in set *x*.

According to Eq. (3) and Eq. (4), the probability of the walker teleporting from *u* to *Y*_*j*_, denoted as *p*(*u* → *Y*_*j*_) is calculated as Eq. (5).


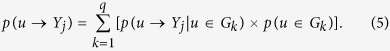


The algorithm to calculate the probability of a node belonging to each community is described in Table 3. A community is expanded by iteratively adding the node which is the most likely to belong to the community.

### Community optimization

Each node in a connected network should be involved into a community, but several nodes with very low degree may still be not included in any communities. In other words, the node which is not added into a communities always has small number of neighbors. Given the node *u* which is not added into any communities and the community *Y*_*k*_, denoting by *T*(*u, Y*_*k*_) that the tightness between the node *u* and the community *Y*_*k*_(1 ≤ *k* ≤ *q*), we have


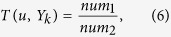


where *num*_1_ denotes the number of nodes which have connections with the node *u* in the community *Y*_*k*_, and *num*_2_ is the number of nodes in the community *Y*_*k*_. The node is added to the community which has the largest tightness with it.

Two or more of the expanded communities may have a large number of common nodes. The communities which are expanded from different communities may be identical or similar, in which case the expanded communities should be merged into one community. If two communities *C*_*i*_ and *C*_*j*_ satisfy the following formula, then they can be merged into a larger community *C*.


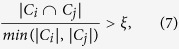


where *ξ* is a threshold. Let *ξ* = 0.5, meaning that most members of the small community are in the large community, the two communities can be merged into one.

### Time complexity

In this section, we analyze the time complexity of the proposed algorithm. The time complexity is *O*(*dN*) to find local maximum degree nodes in a network, where *N* is the number of nodes in the network and *d* is the average degree of nodes. At the stage of detecting seed communities, the time used to detect seed communities based on local maximum degree nodes is *O*(*dp*), where *p* is the number of local maximum degree nodes. At most, there are *p* seed communities. At the stage of expanding communities, it needs to calculate the probability that a node teleports to each node in communities based on an iterative formula. It takes a time complexity of *O*(*logm*) in each iteration as stated in ref. 32, where *m* is the number of nodes in the communities. The worst-case complexity is *O*(*logN*). The time complexity of the stage after *p* iterations is *O*(*plogN*). At the stage of community optimization, a small number of nodes which are not in any communities needs to be added to a community based on the tightness. The time complexity is *O*(*ph*) to calculate the tightness between a node and a community, where *h* represents the number of nodes which are not in any communities. Therefore, the time complexity of the entire algorithm is *O*((*d* + *p*)*N*), since *O*(*dp*) = *O*(*dN*), *O*(*ph*) = *O*(*pN*) and *O*(*p*log*N*) < *O*(*pN*).

### Calculation of similarity

We calculate the similarity between the nodes *v*_*i*_ ∈ *V* and *v*_*j*_ ∈ *V* (1 ≤ *i, j* ≤ *m*) as follows^33^.


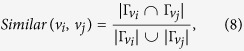


where 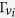 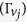 is the neighborhood of *v*_*i*_ (*v*_*j*_) in a network, and |*x*| indicates the cardinality (i.e., number of elements in) the set *x*.

In our work, the similarity between nodes is used to calculate the matrix *M* and the initial probability vector *s*_0_. The similarity between nodes is normalized to obtain the matrix *M*, i.e., 
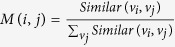
. Let *v*_*j*_ = *u* in Eq. (8). The similarity between nodes *u* and *v*_*i*_ ∈ *V* (1 ≤ *i* ≤ *m*) is calculated, and it is denoted as *Similar*(*v*_*i*_, *u*) (*Similar*(*v*_*i*_) for short). The initial probability vector *s*_0_ is the normalization of vector *Similar*(*v*_*i*_) (i.e., 
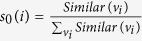
).

### Evaluation measures

For networks whose true partitions are known, Normalized Mutual Information (*NMI*)^34^ and the F-measure (*F*1)^14^ are widely used indexes for measuring the performance of community detection algorithms^1^,^35^,^36^. Both of them reflect the detection results from different points of view. Thus, both *NMI* and *F*1 are employed here as indexes to test the detection results.

*NMI* is defined as follows:


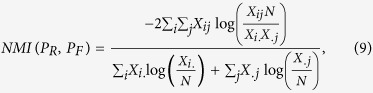


where *N* is the number of nodes, *X* is a 2 × 2 matrix with *X*_*ij*_ being the number of nodes from the real community *i* that also belong to the found community *j, X*_.*j*_ = *X*_1*j*_ + *X*_2*j*_, and *X*_*i*._ = *X*_*i*1_ + *X*_*i*2_. If the partitioning result *P*_*F*_ is the same as *P*_*R*_, then *NMI*(*P*_*R*_, *P*_*F*_) = 1; if they are completely opposite, then *NMI*(*P*_*R*_, *P*_*F*_) = 0.

The *precision* is the ratio of the number of identified nodes which belong to the true community and the number of nodes in a discovered community^14^. The *recall* is the fraction of identified nodes which belong to the true community in the true community^14^. *F*1 is the combination of the *precision* and the *recall*, and it is calculated as follows:


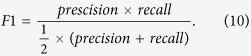


The *precision* and the *recall* only reflect one aspect of the performance of an algorithm. However, *F*1 is the combination of *precision* and *recall*, and it takes the performance of an algorithm into a comprehensive consideration. Therefore, *F*1 is of more comparative significance, compared with *precision* and *recall*.

## Additional Information

**How to cite this article:** Su, Y. *et al*. A seed-expanding method based on random walks for community detection in networks with ambiguous community structures. *Sci. Rep.* **7**, 41830; doi: 10.1038/srep41830 (2017).

**Publisher's note:** Springer Nature remains neutral with regard to jurisdictional claims in published maps and institutional affiliations.

## Figures and Tables

**Table 1:** The number of strong and weak communities in real-world networks.

	Karate	Dolphins	Polbooks	Football
strong	0	1	1	8
weak	2	1	2	4

‘Karate’, ‘Dolphins’, ‘Polbooks’ and ‘Football’ represent the Zachary’s Karate Club network, the Bottlenose Dolphins network, the Books about US politics network and the American College Football network, respectively.

**Table 2:** The performance in strong and weak communities of real-world networks.

	Karate		Dolphins		Polbooks		Football		Total	
	s(0)	w(2)	s(1)	w(1)	s(1)	w(2)	s(8)	w(4)	s(10)	w(9)
GN	0	0	0	1	0	0	1	0	10%	11.11%
FN	0	0	0	0	0	0	1	0	10%	0
FUA	0	2	0	0	0	0	0	0	0	22.22%
FEC	0	0	0	0	0	0	3	2	30%	22.22%
LMDR	0	0	1	0	0	0	5	1	60%	11.11%
RWA	0	2	1	1	1	1	5	3	60%	77.78%

‘Karate’, ‘Dolphins’, ‘Polbooks’ and ‘Football’ represent the Zachary’s Karate Club network, the Bottlenose Dolphins network, the Books about US politics network and the American College Football network, respectively. ‘s(*)’ represents the number of strong communities is ‘*’ in a specific network. ‘w(**)’ represents the number of strong communities is ‘**’. ‘Total s(10)’ means the total number of strong communities in four real-world networks is 10, and ‘Total w(9)’ means the total number of weak communities in four real-world networks is 9.

**Table 3:** The algorithm to calculate the probability of a node belonging to each community.

*Input*	Node-set *V* = {*v*_1_, …, *v*_*m*_}, a node *u* and the set of communities *Y* = {*Y*_1_, …, *Y*_*q*_}, where *v*_*i*_ represent the node included in a community, and *q* is the number of communities.
*Output*	The probability vector for the node *u* in each community *P*(*u* → *Y*) = (*p*(*u* → *Y*_1_), …, *p*(*u* → *Y*_*q*_))
Step 1	Initialize an array *PC* with dimension of *m* × *q* (Save the conditional probability that the walker teleports from the node *u* to the node *v*_*i*_ when the node *u* belongs to the *k*^*th*^ community); Initialize an array *PP* with dimension of *q* × 1 (Save the probability for the node *u* in the community *G*_*k*_).
Step 2	For *k* = 1 to *q* do
Step 3	Construct the graph *G*_*k*_;
Step 4	Calculate the matrix *M* and the initial vector *s*_0_;
Step 5	Iterate the Eq. (2) until *s* is convergent, and the probability vector *π* = (*π*_1_, …, *π*_*m*_) is *s*;
Step 6	Calculate *p*(*u* → *Y*_*i*_|*u* ∈ *G*_*k*_) 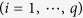 , and then *PC*(*k, i*) = *p*(*u* → *Y*_*i*_|*u* ∈ *G*_*k*_);
Step 7	Calculate *p*(*u* ∈ *G*_*k*_), and then *PP*(*k*) = *p*(*u* ∈ *G*_*k*_);
Step 8	End For
Step 9	Normalize *PC* and *PP*; Calculate *p*(*u* → *Y*_*i*_): *p*(*u* → *Y*_*i*_) = *PC* × *PP*;
Step 10	Return *P*(*u* → *Y*) = (*p*(*u* → *Y*_1_), …, *p*(*u* → *Y*_*q*_)).
